# Flexural Behaviour of GFRP-Softwood Sandwich Panels for Prefabricated Building Construction

**DOI:** 10.3390/polym15092102

**Published:** 2023-04-28

**Authors:** Ahmed D. Almutairi, Yu Bai, Wahid Ferdous

**Affiliations:** 1 Department of Civil Engineering, College of Engineering, Qassim University, Unaizah 56452, Saudi Arabia; 2 Department of Civil Engineering, Monash University, Clayton, VIC 3800, Australia; yu.bai@monash.edu; 3 Centre for Future Materials (CFM), School of Engineering, University of Southern Queensland, Toowoomba, QLD 4350, Australia; wahid.ferdous@usq.edu.au

**Keywords:** fibre-reinforced polymer, softwood timber, mechanical properties, adhesive bonding, sandwich structures

## Abstract

Studies have shown that the proper selection of core materials in sandwich structures improves the overall structural performance in terms of bending stiffness and strength. The core materials used in such systems, such as foam, corrugated, and honeycomb, are frequently applied in aerospace engineering. However, they are a costly option for civil engineering applications. This paper investigates the bending performance of the proposed GFRP softwood sandwich beams assembled using pultruded GFRP with adhesive connection methods for potential applications in prefabricated building construction. The ultimate load capacity, load–deflection responses, failure modes, bending stiffness, load–axial-strain behaviour, and degree of composite action were experimentally evaluated. The effects of varying shear-span-to-depth ratios a/d between 2 and 6.5, as well as different timber fibre directions of the softwood core, on the overall structural performance were clarified. The results showed that changing the timber fibres’ orientation from vertical to longitudinal shifted the failure mode from a brittle to progressive process. Moreover, the adhesive bonding was able to provide full composite action until the failure occurred. Finally, numerical modelling was developed to understand failure loads, deformation, failure modes, and strain responses, and to evaluate bending stiffness and composite action. The results showed satisfactory agreement with the experiments.

## 1. Introduction

FRPs or fibre-reinforced polymers have been commonly and increasingly used in the past few decades as strengthening systems made of composite materials to repair existing structural elements such as timber, steel, and concrete or as load-carrying structural members, for example, sandwich structural systems [1,2,3,4,5,6,7,8,9]. The use of GFRP or glass-fibre-reinforced polymer has particularly been growing due to its substantial advantages such as ease of installation, resistance to corrosion, high strength-to-weight ratio, and, most importantly, cost-effectiveness when compared with other composite materials such as CFRPs or carbon-fibre-reinforced polymers [10,11,12,13,14,15,16,17,18,19]. Sandwich structures have been increasingly used in civil engineering applications, which are a type of structure made of two fabricated GFRP face sheets and bonded to a lightweight material called a core [20]. In such structure systems, the face sheet layers provide an enhancement in the strength and bending stiffness, while the core provides the majority of the shear stiffness [21,22,23,24,25]. FRP web-flange sandwich structures have been successfully used in the applications of bridge deck construction [26,27,28,29]. Several studies have shown that selecting the proper type of core materials in FRP web-flange sandwich structures enhances the overall structural performance in terms of stiffness and strength, as well as prevents out-of-plane buckling of the face sheets [30,31,32,33]. Core materials used in such systems, such as foam, corrugated, and honeycomb, are frequently applied in aerospace engineering [34,35]. However, they are a costly option for civil engineering applications.

Low-cost and high-performance solid cores could address this problem in civil engineering applications. In recent years, wood-based cores have attracted attention for their many benefits. Wood is a strong and durable material that can withstand high loads and pressures. It provides excellent structural strength when used as a core material in sandwich panels [36,37,38]. Their light weight makes them more convenient than other materials, such as metals or concrete. Consequently, wood core sandwich panels are lighter, making them easier to transport and install [39]. Aside from this, wood is an excellent natural insulator that provides good thermal insulation. Using sandwich panels with a wood core can help regulate temperatures, reducing energy consumption [40]. Additionally, wood has good acoustic insulation properties, making sandwich panels with a wood core ideal for sound-proofing buildings. Most importantly, wood is a renewable resource, and its use during construction helps promote sustainability. Consequently, wood core sandwich panels have a lower carbon footprint than traditional sandwich panels. Overall, sandwich panels that use wood as their core material can provide excellent strength and thermal and acoustic insulation, and improve environmental sustainability [41,42]. Considering these advantages, wood core sandwich panels could be a suitable option for a wide range of uses, including building construction, transportation, the maritime industry, and aerospace. This study therefore investigates the mechanical performance of sandwich structures comprising pultruded GFRP panels as face sheets and lightweight Australian Radiata pine softwoods as core material that has not yet been well studied despite having great potential. 

In this study, the sandwich panels were manufactured from Australian Radiata pine softwood as the core material and pultruded-glass-fibre-reinforced polymer (GFRP) as the face sheets. The bending performance of such developed GFRP softwood sandwich beams was focused. The geometries of the sandwich beams were designed in a way to allow easy assembly by connecting the face panel onto softwoods through adhesive bonding. Experimental results on the failure modes, load-bearing capacities, load–displacement curves, and load–strain responses were obtained and discussed. The effects of different timber fibre directions as well as varying shear-span-to-depth ratios on the overall structural performance were clarified, and the degree of composite action was further investigated and understood based on the measured strain results. Finally, finite element (FE) models were developed to describe the deformation, strain responses, and failure loads, and to evaluate composite action and structural stiffness. The novelty of this study was the development of a high-performance sandwich panel at a reasonable price. A key scientific challenge of this panel is its ability to remain functional under structural loads as the modulus of elasticity varies between the skins and the core. The outcome of this study will help us understand the feasibility of the sandwich concept and can benefit civil engineers in the build-out of sustainable infrastructure for the future.

## 2. Experimental Program

### 2.1. Materials

**Figure 1:**
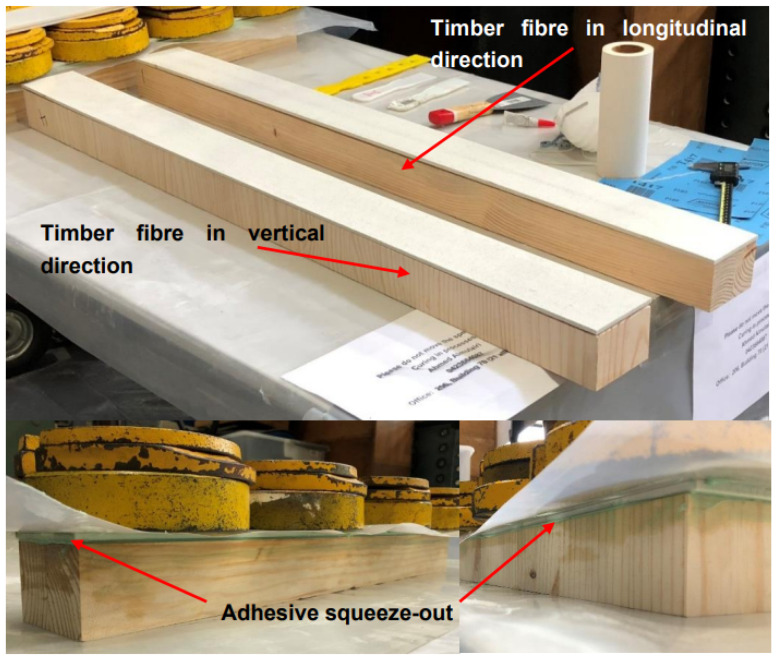
GFRP-softwood sandwich beam during fabrication.

The proposed sandwich beams consisted of pultruded-glass-fibre-reinforced polymer (GFRP) composites which were supplied by Exel Composites, Australia, as the face sheets that were adhesively bonded to a softwood core. The epoxy adhesive bond used in the fabrication process was two components of epoxy (BPR135G3/BPH137G). The core material of the sandwich beam specimens was unseasoned softwood timber called (*Pinus radiata*) or radiata pine, a plantation species that is widely grown in Victoria, Australia. The softwood cores were made with two different fibre directions as vertical or longitudinal grain, as shown Figure 1. 

The longitudinal grain represents the timber fibre direction in parallel with GFRP fibres, as well as the span direction of sandwich beams. The vertical grain represents the timber fibre direction perpendicular to the GFRP fibres’ direction or the span direction. Using the Glulam (glued laminated timber) technique, the softwood core was manufactured as individual layers based on the proposed grain directions, which were assembled using a structural adhesive. Small blocks with dimensions of (60 mm × 90 mm × 45 mm) adhered together with wood grain in the vertical direction to form the required span. For the core with longitudinal grain, two pieces of timber with dimensions of (45 mm × 60 mm × required span) were glued together. The advantages of the core manufacturing approach are to minimise the variability in the properties of timber as naturally grown material [43] and allowing dimensional flexibility beyond the natural size of timber. The material properties of the proposed GFRP-softwood sandwich beams components are summarised in Table 1. The softwood timber material properties were tested and obtained according to the ASTM D143-94 standard [44]. The GFRP face sheets were cut from a pultruded flat panel along the fibre direction. According to [31], the pultruded GFRP material consisted of E-glass fibres with 47.8% in volume embedded in polyester resin. The fibre architecture comprised one unidirectional roving layer between two mat layers. The modulus and strength properties of the GFRP obtained according to relevant standards and methods [45,46,47] are summarised in Table 1. Under the tensile test in accordance with ASTM D638 [48], the epoxy adhesive properties were obtained and are presented in Table 1. 

### 2.2. Specimen Details and Preparation

**Figure 2:**
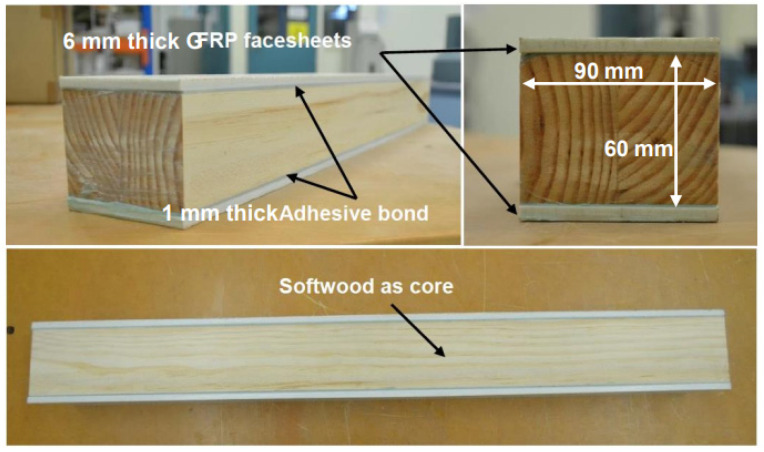
Fabricated specimen of the proposed sandwich beams.

**Figure 3:**
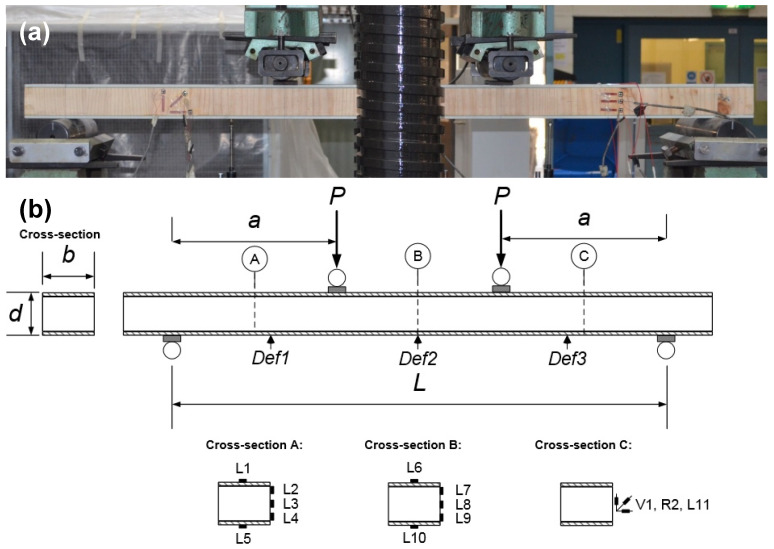
(**a**) Experimental setup of V6.5; (**b**) LVDT instrumentation and strain gauge.

The 6 mm thick pultruded GFRP flat panels as face sheets were prepared by cutting the panels into the required dimensions using a water jet cutter. The softwood cores were 90 mm in width and 60 mm in depth. A 1 mm thick layer of epoxy adhesive was used to join the face sheet and core together. The adhesive layer was controlled by using 1 mm diameter spacers between the two materials (GFRP and softwood). In order to secure the bond quality (and therefore a satisfactory composite action), all adherents were cleaned by acetone after sanding and degreasing all surfaces in accordance with the procedure outlined in the Eurocomp design code and handbook [49]. The softwood core was first bonded to the top face sheet and allowed to cure for four days before the application of the bottom face sheet. Weights were used to provide pressure while the adhesive cured for 14 days at room temperature (see Figure 1). The fabricated specimens after curing are shown in Figure 2 where the longitudinal fibre direction can be seen. The dimensions of the overall cross-section of the sandwich beam were the same for all the specimens, as shown in Figure 3b, where d (74 mm) is the overall sandwich beam depth and b (90 mm) is the sandwich beam width. The other parameters P, a, and L are the applied load, shear span, and span of the tested beam, respectively. The specimens had shear-span-to-depth ratios a/d of 2, 4, and 6.5 and they are labelled with a letter followed by a number in subscript, where the letter represents the fibre direction of the timber core, i.e., L for longitudinal and V for vertical; the number in subscript represents the shear-span-to-depth ratio a/d. The geometric parameters of each of the GFRP-softwood beam specimens are listed in Table 2.

### 2.3. Experimental Setup and Instrumentation

The sandwich beam specimens were simply supported and tested under four-point static bending in accordance with ASTM C393 [50], as shown in Figure 3a. A bending load on the sandwich specimens was implemented using a Baldwin 500 kN testing machine for the specimens with an a/d of 2 or a 250 kN Amsler machine for the other specimens. Rubber pads were used under the loading points to distribute the load and prevent local crushing. A displacement control mode was adopted during the loading processes at a rate of 0.5, 1, or 2 mm/min corresponding to shear-span-to-depth ratios a/d of 2, 4, or 6.5, respectively. The deflections were measured using three linear potentiometers (linear variable differential transformers—LVDTs), as shown in Figure 3b. Strain gauges were installed in the longitudinal direction along the depth of each specimen, as shown in cross-sections A and B in Figure 3b, to investigate the degree of composite action there. Strain gauges as shown in cross-section C in Figure 3b were positioned to represent a point halfway between the support and the point load and, hence, measure the strain in the region of maximum shear. All strain gauges were checked with an ohmmeter prior to the testing to ensure their accuracy and functionality.

## 3. FE Modelling

**Figure 4:**
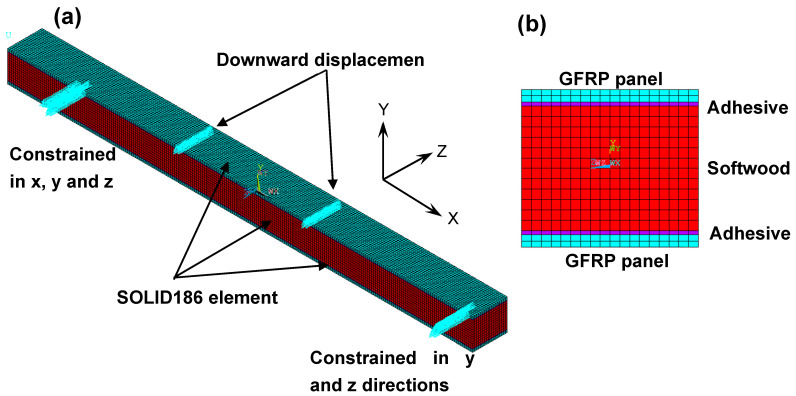
FE modelling of specimen L4: (**a**) overall model with boundary conditions; (**b**) cross-sectional views of specimen L4.

FE modelling was conducted using Ansys in order to describe and simulate the mechanical responses of the sandwich beam specimens made of GFRP-softwood in terms of strain responses, load vs. deflection, and failure modes, as well as the composite action between the softwood core and GFRP panels. Three-dimensional (3D) models were established according to the detailed dimensions of the sandwich beams made of GFRP-softwood, and Figure 4a shows an example of specimen L4. Y and Z axes were defined in the coordinate system to represent the cross-sectional plane of the GFRP-softwood sandwich beams, where the span direction represents the X axis of the modelled GFRP-softwood specimens. The adhesive, GFRP, and timber were modelled using Solid186 in the FE model as a higher-order, 3D, 20-node brick-like element where all edges have a mid-node, which is capable of supporting a large strain value along with large deflection. The element size was controlled to be a maximum of 5 mm in the model, where this size of the element was capable of providing stable and accurate modelling results. 

The GFRP and timber materials were defined as linear elastic orthotropic, as shown in Figure 4b, with the modulus properties listed in Table 1. The epoxy adhesive was modelled as an isotropic linear elastic material (see Figure 4b) with the shear and elastic modulus given in Table 1. The FE model was subjected to a simply supported boundary condition where the nodes of the specimens at the support locations were constrained in the X, Y, and Z directions at one end, and the Y and Z directions at the other end where all nodes of the degree of freedom in X are free as commonly practiced [51]. In the Y direction, the translational degree of freedom at the top nodes was allowed at the position of loading, and a vertical downward load was applied at loading locations in the form of displacement, as shown in Figure 4a. In the solution process, the results such as the reaction forces at the locations of supports as well as the mid-span deflection were recorded in order to perform a comparison with experimental results. At the LDVT locations, the deflections were recorded as well in order to capture the deflection shapes. When any stress component at a certain location exceeded the corresponding given material strength, the ultimate loads of the modelled specimens were then determined in Table 1.

## 4. Results and Discussion

### 4.1. Failure Modes

**Figure 5:**
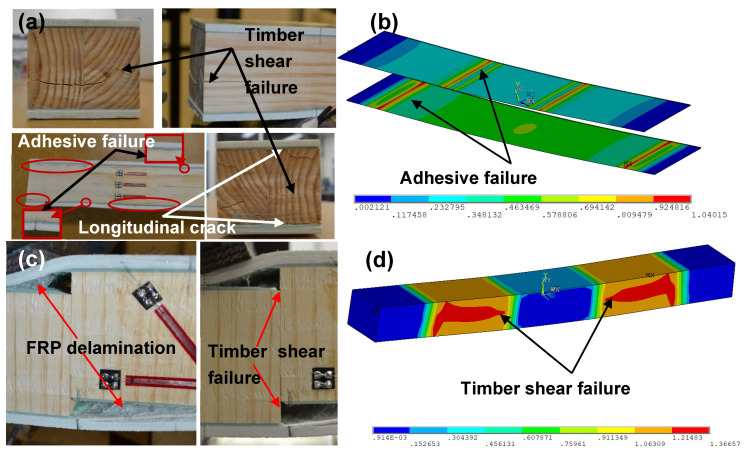
Failure modes on the specimens with a/d of 2: (**a**) failure modes of specimen L2 with longitudinal timber fibre direction; (**b**) failure modes for modelled specimen L2; (**c**) failure modes of specimen V2 with vertical timber fibre direction; (**d**) failure modes for modelled specimen V2.

Depending on the different timber fibre direction of the core as well as a varying shear-span-to-depth ratio a/d of the six GFRP-softwood sandwich specimens, the experimental investigation exhibited different failure modes. The specimen with a shear-span-to-depth ratio a/d of 2 and longitudinal timber fibre direction (L2) was failed progressively. At 98 kN, shear failure at the timber core occurred close to the neutral axis N.A where the maximum shear stress was expected to occur. When the maximum load capacity of 101 kN was reached, the adhesive failed at the load and support locations, causing a longitudinal crack of the timber in the top and the bottom of the core, as shown in Figure 5a.

**Figure 6:**
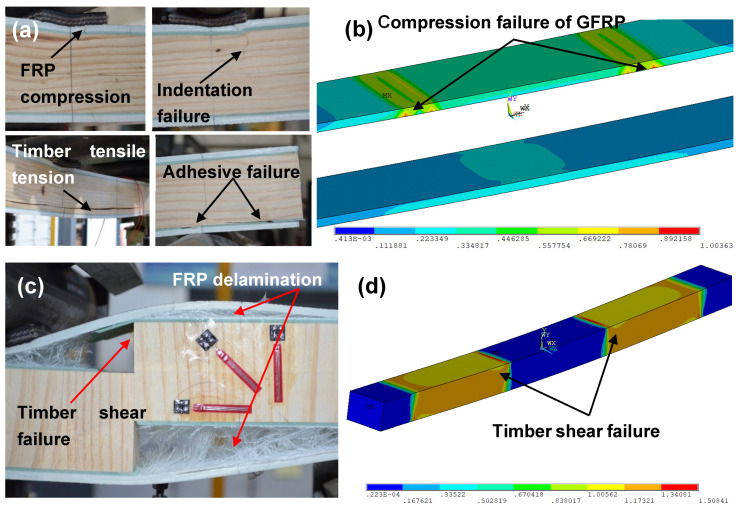
Failure modes on the specimens with a/d of 4: (**a**) failure modes of specimen (L4) with longitudinal timber fibre direction; (**b**) failure modes for modelled specimen (L4); (**c**) failure modes of specimen (V4) with vertical timber fibre direction; (**d**) failure modes for modelled specimen (V4).

**Figure 7:**
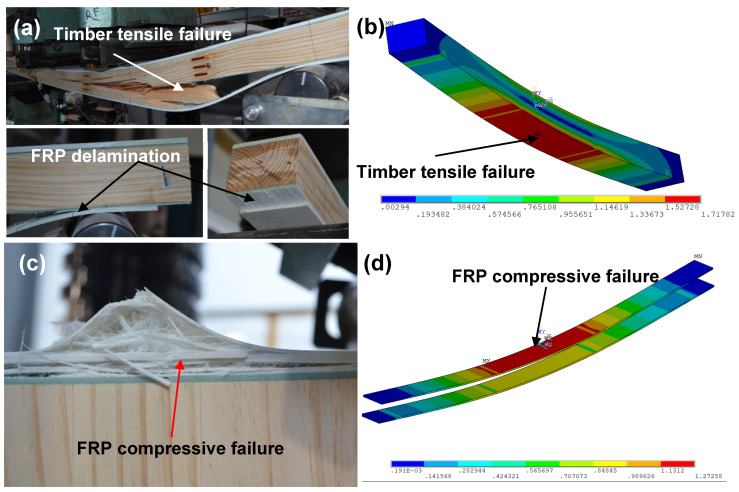
Failure modes on the specimens with a/d of 6.5: (**a**) failure modes of specimen (L6.5) with longitudinal timber fibre direction; (**b**) failure modes for modelled specimen (L6.5); (**c**) failure modes of specimen (V6.5) with vertical timber fibre direction; (**d**) failure modes for modelled specimen (V6.5).

For specimen L4 with an a/d of 4, progressive failure was observed, resulting in a gradual decrease in load and stiffness, as seen in Figure 6a. The first failure was GFRP compressive failure at 84 kN under the loading points, which was immediately after the elastic region, leading to indentation failure of the timber core due to the high local stress; this was followed by timber tensile failure at the bottom of the core between the loading points. Figure 7a shows specimen L6.5 with an a/d of 6.5 and a bending sudden failure of the timber core at the maximum load capacity of 68 kN, which causes delamination failure of GFRP. The specimens of a/d of 2 and 4 with vertical timber fibre directions V2 and V4 showed similar failure behaviours (see Figure 5c and Figure 6c) at their maximum load capacities of 64.3 kN and 53.9 kN for specimens V2 and V4, respectively, where sudden shear failures were observed on the timber core between the loading point and the support. Likewise, this also caused delamination failure of the GFRP.

For specimen V6.5 with an a/d of 6.5, the failure was sudden at the maximum load capacity of 37.1 kN, which was governed by GFRP compressive failure presented in Figure 7c. The failure modes of the specimens from the FE analysis are provided in Figure 5, Figure 6 and Figure 7. In these figures, the colours change gradually from blue to red where the red colour indicates the failure locations when any stress component at those locations exceeds the corresponding material strength given in Table 1. The results showed that sandwich beams with an a/d of 2 failed in shear and adhesive failure. Combined failure modes of shear in timber and compression in GFRPs were seen for specimens L4 and V4 with an a/d of 4. Bending failure in timber and compressive failure in GFRPs were observed for specimens L6.5 and V6.5 with an a/d of 6.5. From the FE modelling results, the failure modes of the tested specimens can be described reasonably well.

### 4.2. Load–Displacement Behaviour

**Figure 8:**
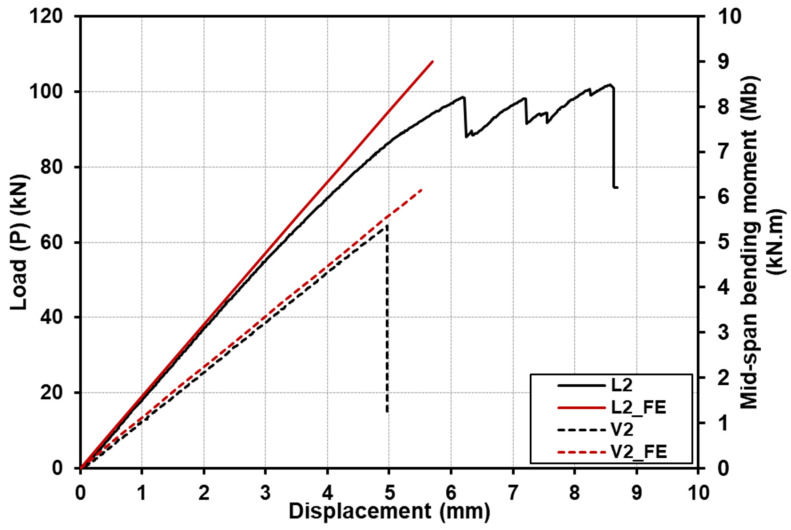
Comparison of deflection responses with load and bending moment for specimens with a shear-span-to-depth ratio a/d of 2, from both experiments and FE analysis.

**Figure 9:**
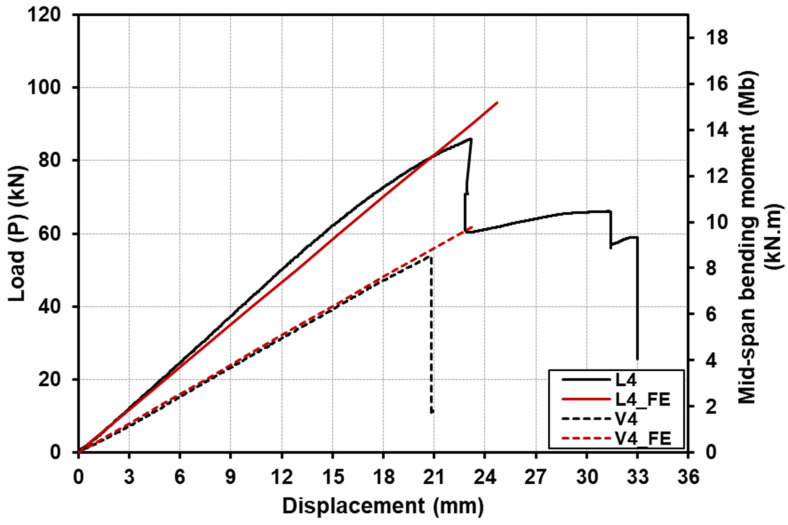
Comparison of deflection responses with load and bending moment for specimens with a shear-span-to-depth ratio a/d of 4, from both experiments and FE analysis.

**Figure 10:**
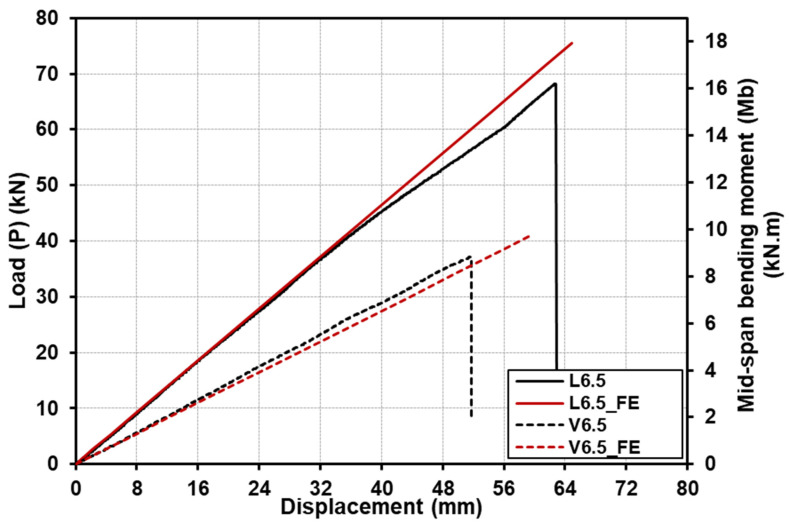
Comparison of deflection responses with load and bending moment for specimens with a shear-span-to-depth ratio a/d of 6.5, from both experiments and FE analysis.

Figure 8 shows the comparison for the load (or moment)–deflection responses up to the failure of the specimens with a shear-span-to-depth ratio a/d of 2. The load (or moment)–deflection responses for specimens with a shear-span-to-depth ratio a/d of 4 are shown in Figure 9, whereas Figure 10 illustrates the load (or moment)–deflection responses for specimens with a shear-span-to-depth ratio a/d of 6.5. From the mentioned figures, the load (or moment)–deflection curves for the specimens with longitudinal-timber-fibre-direction cores are presented with continuous solid black lines, where the curves for the specimens with vertical-timber-fibre-direction cores are in dashed black lines. 

Specimen L2 with a shear-span-to-depth ratio a/d of 2 and longitudinal timber fibre direction showed approximately linear responses for load (or moment) and deflection until the first failure occurred, as shown in Figure 8. After the first failure, several drops were observed due to progressive failure of different modes (such as timber shear and adhesive failures). The first drop of the load was at 98 kN due to timber shear. However, the load increased again with almost the same stiffness prior to the first drop. At almost the same load before the first load drop, the second drop of the load was observed, and the load increased again with almost the same stiffness before the first and the second drops; the last load drop occurred due to adhesive failure at the ultimate load of 101.7 kN. The maximum moment capacity of the tested specimens can be calculated from Equation (1), where *M_b_* is the moment capacity of the GFRP-softwood sandwich specimen in bending, *Pu* is the ultimate load of the tested specimen, and *a* is the shear span. The results are presented in Table 3 and the maximum moment capacity of L2 was 8.4 kN·m.

(1) $$M_{b}=\frac{P_{u}\;a\;}{2}$$

Specimen V2 with vertical timber fibre direction exhibited a linear load–deflection behaviour, as shown in Figure 8, until sudden brittle failure occurred, where the ultimate load was 64.3 kN and the moment capacity was 5.3 kN·m. 

For specimen L4 with a shear-span-to-depth ratio a/d of 4 and longitudinal timber fibre direction, the load–deflection response was approximately linear, as shown in Figure 9, until the first failure occurred. The specimen developed progressive failure with three significant drops, leading to a decrease in the load and stiffness gradually. The first drop of the load was due to GFRP compressive failure under the loading points at the ultimate load of 86 kN (moment of 13.5 kN·m). The load increased again until reaching 65 kN, where the second drop of the load occurred due to indentation failure of the timber core caused by the high local stress at the loading points. The last drop occurred at 58 kN due to tensile failure of the softwood core. In contrast to specimen V4 with vertical timber fibre direction, the load–deflection behaviour was linear until brittle sudden failure occurred at the ultimate load of 53.9 kN and moment capacity of 8.5 kN·m.

**Figure 11:**
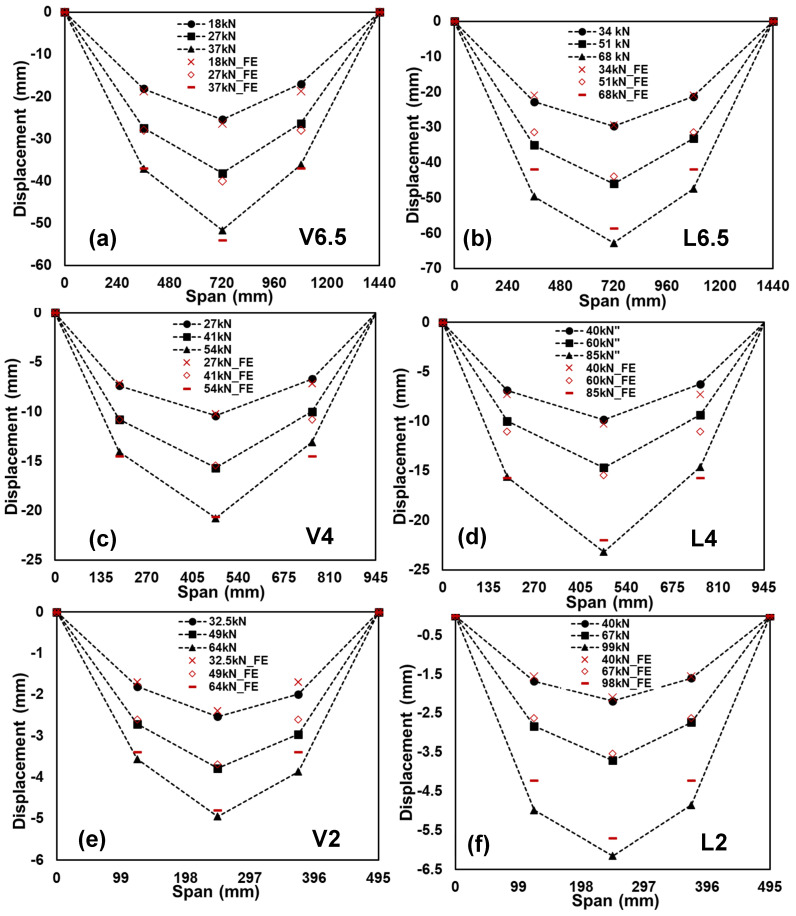
Comparison of experimental and FE analysis of deformed shapes for the tested sandwich beam specimens: (**a**) V6.5; (**b**) L6.5; (**c**) V4; (**d**) L4; (**e**) V2; (**f**) L2.

The GFRP-softwood sandwich specimens with a shear-span-to-depth ratio a/d of 6.5 for longitudinal or vertical timber fibre direction, L6.5 or V6.5, exhibited similar load–deflection behaviours, as shown in Figure 10, which were linear until sudden brittle failure occurred at their ultimate loads. The ultimate load for L6.5 and V6.5 was 68.3 kN and 37.1 kN, respectively, where the corresponding maximum moment capacity was 16.4 kN·m and 8.9 kN·m for L6.5 and V6.5, respectively. The deformed shapes for the tested sandwich specimens were measured along the span, as shown in Figure 3b. Figure 11 illustrates the deformation shape for all the tested specimens at the different load levels. All the tested sandwich beam specimens showed a stable and gradual increase in displacement with the increase in load and also indicated that beam theory may be applicable in this study.

The load (or moment)–deflection responses of the GFRP-softwood sandwich beam specimens were obtained from the FE analysis, and the comparisons with the experimental results are presented in Figure 8, Figure 9 and Figure 10. The FE results are presented as continuous solid red lines for the specimens with longitudinal-timber-fibre-direction cores, while the specimens with vertical-timber-fibre-direction cores are presented as dashed red lines. The load (or moment)–deflection curves from the FE analysis showed good agreement with experimental results, especially for the linear developments in the initial stages. Overall, as seen in Table 3, the comparisons of the results of failure loads that were obtained from the FE analysis were satisfactorily compared to the experimental results with a maximum difference of 12.2%. Furthermore, the deformation shapes of the modelled specimens compared well with the experimental results at the same load levels, as presented in Figure 11.

### 4.3. Load–Strain Behaviour and Composite Action

**Figure 12:**
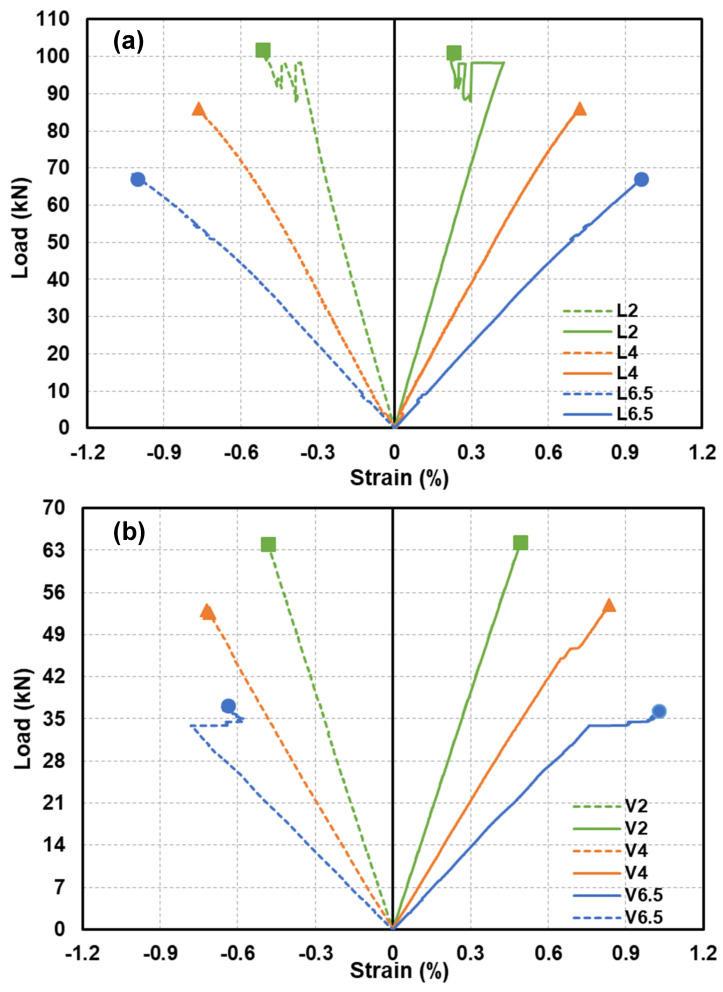
Load–axial-strain responses at mid-span for experimental specimens: (**a**) with longitudinal-timber-fibre-direction cores; (**b**) with vertical-timber-fibre-direction cores.

The load–axial-strain curves at the mid-span are shown in Figure 12a,b for the tested GFRP-softwood sandwich specimens. At the mid-span, the strain values were measured by strain gauges that were attached corresponding to the positions as marked in Figure 3b (Section B). Overall, the load–strain curves, presented in Figure 12a for the specimens with longitudinal-timber-fibre-direction cores and illustrated in Figure 12b for the specimens with vertical-timber-fibre-direction cores, showed linear behaviour up to the ultimate loads. These could indicate that satisfactory bonding quality was maintained between the GFRP panels and softwood core at the mid-span section during the loading process for all tested specimens. The ultimate strain values at the mid-span top (compression) and bottom (tension) of GFRP panels for all tested are listed in Table 4.

It can be noticed that the ultimate strain magnitudes in both tension and compression sides increased when the shear-span-to-depth ratio a/d increased, and this was caused by the deflection developments when the shear-span-to-depth ratio a/d increased. Comparisons of the ultimate strains in the FE and experimental results for all the modelled specimen scenarios are presented in Table 4. The comparison of the bending strains at the bottom and top that were located at the mid-span, which were obtained from the FE analysis, was satisfactory as compared to the experimental results, with deviations ranging from −15.2% to 10.7%.

**Figure 13:**
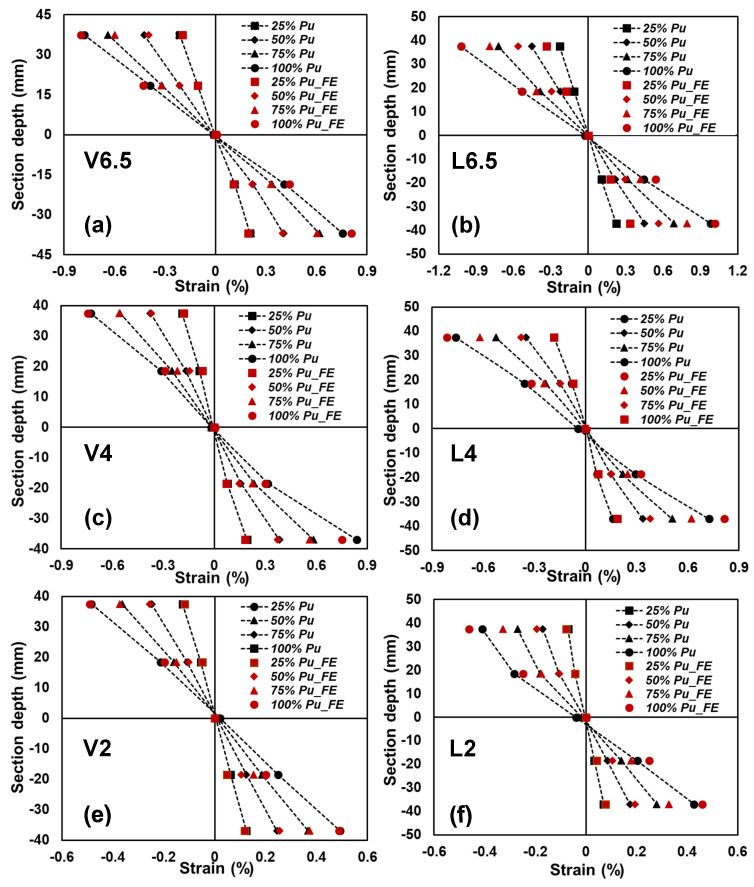
Comparison of experimental and FE axial strain distributions along the depth of a cross-section at mid-span for the tested sandwich beams specimens: (**a**) V6.5; (**b**) L6.5; (**c**) V4; (**d**) L4; (**e**) V2; (**f**) L2.

The axial strain distributions at the mid-span along the section depth of all the tested GFRP-softwood sandwich specimens at different load levels are shown in Figure 13. All scenarios exhibited similar behaviour, where a linear axial strain distribution from the upper GFRP panel through the depth of the softwood core to the lower panel at the midspan was observed. Such results may indicate that full composite action between the bonded GFRP and timber was achieved at the mid-span. The full composite action was maintained at the mid-span for the tested specimens until the first failure occurred, as evidenced by the linear strain distributions along the depth. The strain distribution from FE modelling is also provided in Figure 13 as separate red points, and satisfactory agreements with the experimental results can be seen.

**Figure 14:**
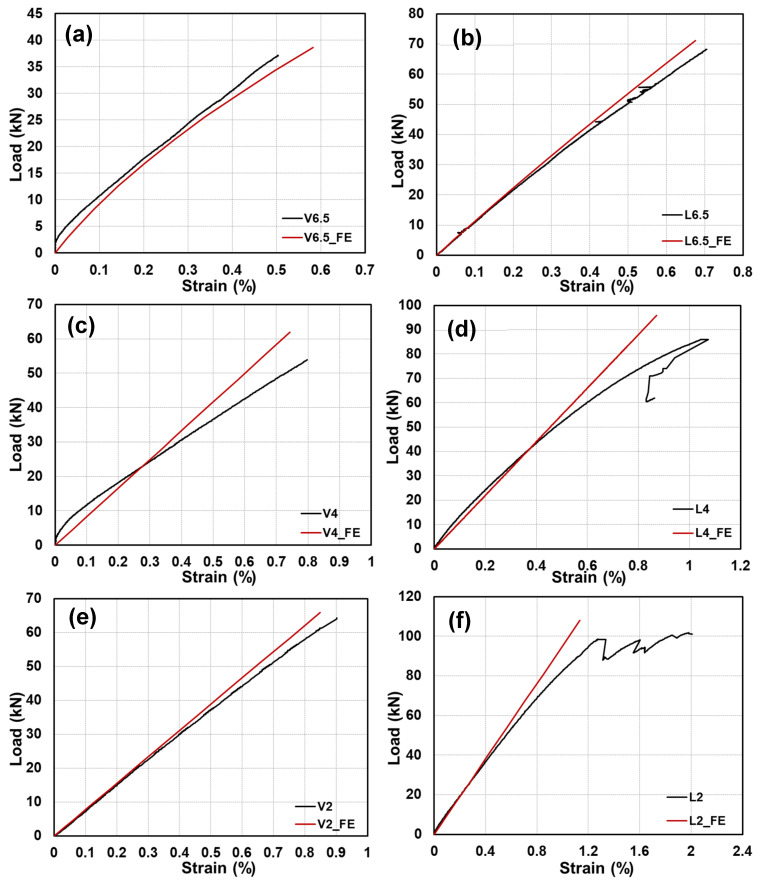
Comparison of experimental and FE shear strains at shear-span for tested sandwich specimens: (**a**) V6.5; (**b**) L6.5; (**c**) V4; (**d**) L4; (**e**) V2; (**f**) L2.

Due to the difference in shear-span-to-depth ratio a/d as well as the varying timber fibre direction of the core, strain gauges were attached at the shear span of the GFRP-softwood sandwich specimens corresponding to the locations marked in Figure 3b (Section C). The shear strain was determined from Equation (2) by using the vertical, horizontal, and diagonal strain readings.

(2) $$\gamma =2\varepsilon_{d}-\varepsilon_{v}-\varepsilon_{h}$$

where *γ* is the shear strain; *ε_d_* is the measured strain at the shear span in the diagonal direction; while *ε_v_* and *ε_h_* are the measured strain at the shear span in vertical and horizontal directions, respectively. Figure 14 illustrates the load–shear-strain curves for the tested GFRP-softwood sandwich beam specimens. Specimens L2 and L4 showed linear load–shear-strain behaviour initially up to around 60% to 65% Pu, where slightly nonlinear responses were noticed, as shown in Figure 14d,f. The other scenarios such as V2, V4, V6.5, and L6.5 exhibited similar behaviour where linear load–shear-strain curves were observed up to the failure. 

The ultimate shear strain values at the timber core for all tested specimens are listed in Table 4. It can be noticed that the ultimate shear strain magnitudes were greater for the specimens with a shear-span-to-depth ratio a/d of 2, where shear deformability was also expected to be higher. Thus, the ultimate shear strain values decreased when the shear-span-to-depth ratio a/d increased. Table 4 presents a comparison of the FE and experimental results for the ultimate shear strains from all the modelled specimen scenarios, where a satisfactory agreement was seen with the maximum difference of 16.1%

### 4.4. Effect of Shear-Span-to-Depth Ratio and Timber Fibre Direction of Core

The experimental bending stiffness EI of the GFRP-softwood sandwich specimens can be determined from the linear elastic region of the load–deflection curves results given in Figure 8, Figure 9 and Figure 10. The results were calculated using Timoshenko beam theory with shear deformations taken into account using Equation (3) [52].

(3) $$\delta =\frac{PL^{3}}{24\;EI}\left(\frac{3a}{L}-\frac{4a^{3}}{L^{3}}\right)+\frac{Pa}{GA}$$

where *δ* is the deflection at the mid-span for the simply supported beams under four-point bending, *P* is the applied load, *L* is the span length, *E* is the longitudinal elastic modulus, I is the second moment of area, a is the shear span (the distance from the support to the nearest point load), *A* is the cross-sectional area of the core, and *G* is the in-plane shear modulus of the softwood core. The specimens with longitudinal-timber-fibre-direction cores and shear-span-to-depth ratios a/d of 2 to 6.5 (L2, L4, and L6.5) had a shear modulus *G* value of 1.52 GPa; while the specimens with vertical-timber-fibre-direction cores (V2, V4, and V6.5) had a shear modulus *G* value of 0.75 GPa. Both shear modulus *G* values were determined from shear tests on the small clear specimens of softwood according to the ASTM D143-94 standard [44] (see Section 2.1 for details). 

The shear deformability for all the tested GFRP-softwood sandwich specimens can be determined from this term (Pa/GA) of Equation (3), and the results are listed in Table 3. It can be found that the shear deformability is more noticeable for the sandwich beams that have smaller shear-span-to-depth ratios. Thus, the shear deformability for the sandwich beams with a shear-span-to-depth ratio of 2 was greatest among the other specimens. As presented in Table 3, the percentage of deformation due to shear was 38.3% to 54.3% for specimens with a shear-span-to-depth ratio of 2. The shear deformation percentage was 17% and 20% for L4 and V4, respectively. The lowest shear deformation percentage was found for the specimens with a longer span, which had a shear-span-to-depth ratio of 6.5. In those specimens, the shear deformability contributed only 7.3% and 9.2% of the total deformations for specimens L6.5 and V6.5, respectively.

As mentioned above, the experimental bending stiffness EI of the GFRP-softwood sandwich beams can be calculated from Equation (3). The values of P and δ were taken from the linear elastic portions of the experimental load–deflection curves given in Figure 8, Figure 9 and Figure 10, and the results are presented in Table 3. It can be noticed that the bending stiffness for the specimens with longitudinal-timber-fibre-direction cores had slightly higher bending stiffness values, in comparison to the specimens with vertical-timber-fibre-direction cores. This was caused by the values of the tensile and compressive modulus of elasticity, where the softwood core with longitudinal timber fibre direction had a much greater tensile and compressive modulus of elasticity than the specimens with vertical-timber-fibre-direction cores. The FE comparisons with experimental results in bending stiffness for all the modelled specimen scenarios are presented in Table 5. The comparison of bending stiffness obtained from the FE analysis showed satisfactory agreements as compared to the experimental results with the maximum deviation of −8.6%.

## 5. Conclusions

This paper investigated the bending performance of the proposed GFRP softwood sandwich beams assembled using pultruded-glass-fibre-reinforced polymer (GFRP) with adhesive connection methods for potential applications in prefabricated construction. The failure modes, load–deflection responses, ultimate load, bending stiffness, load–axial-strain behaviour, and degree of composite action were experimentally evaluated. The effects of varying shear-span-to-depth ratio a/d, as well as different timber fibre directions of the softwood core, on the overall structural performance were clarified. Moreover, numerical modelling was developed to understand failure loads, deformation, failure modes, and strain responses, and to evaluate bending stiffness and composite action. The following conclusions can be drawn from this study:

- All tested GFRP-softwood sandwich specimens, with the core configurations of either longitudinal or vertical timber fibre direction, exhibited similar load–deflection behaviours, which were approximately linear until the first failure occurred. The increase in moment capacity was similar for the specimens with a shear-span-to-depth ratio a/d of 4 (L4 and V4) by around 60% in comparison to L2 and V2 (a/d of 2). However, there was a different improvement in moment capacity for L6.5 (95%) and V6.5 (68%) in comparison to L2 and V2, respectively.
- The results showed that timber with longitudinal fibre and shear-span-to-depth ratios a/d of 2 and 4 showed different drop patterns due to progressive failure, while that with a shear-span-to-depth ratio a/d of 6.5 showed sudden drop at the ultimate load due to brittle failure. In contrast, the timber core with vertical fibres exhibited sudden failure at the ultimate load for specimens with a/d ratios of 2, 4, and 6.5.
- The strain results showed that the adhesive bonding used in this study was able to provide a full composite action between the GFRP panels and softwood core during the loading process up to failure for all tested specimens. Furthermore, a gradual and stable deformation was observed along the span and depth of each GFRP-softwood sandwich specimen. It can be therefore identified that excellent bonding quality and full composite action were maintained at different load levels along the span.
- The timber fibre direction of the softwood cores used in this study affected the bending stiffness EI of the GFRP-softwood sandwich beams. The specimens with longitudinal-timber-fibre-direction cores had greater bending stiffness in comparison to the specimens with vertical-timber-fibre-direction cores. It was also found that the shear deformability was more pronounced for the sandwich beams that had a smaller shear-span-to-depth ratio, where specimens with a shear-span-to-depth ratio of 2 showed about 38.3% to 54.3% shear deformability.
- The FE modelling approach used in this study for the proposed GFRP-softwood sandwich beams showed results in satisfactory agreement with the experimental results with a maximum deviation of 16.1%. Furthermore, the FE results well described the failure modes and composite action.

Sustainable building materials are becoming increasingly important in the construction industry. These composite sandwich panels can be used to manufacture building floors, walls, and roofs, as well as other structural applications such as composite railway sleepers.

## Figures and Tables

**Table 1:** Material properties of GFRP, softwood timber, and adhesive.

Parameters	Magnitude	Unit
Tensile strength parallel to wood grain direction	58.4	MPa
Tensile strength perpendicular to wood grain direction	4.6	MPa
Compressive strength parallel to wood grain direction	50.4	MPa
Compressive strength perpendicular to wood grain direction	10.6	MPa
Shear strength parallel to wood grain direction	16.5	MPa
Tensile modulus of elasticity parallel to wood grain direction	11.8	GPa
Tensile modulus of elasticity perpendicular to wood grain direction	1.6	GPa
Compressive modulus of elasticity parallel to wood grain direction	10.4	GPa
Compressive modulus of elasticity perpendicular to wood grain direction	1.6	GPa
Shear modulus of elasticity parallel to wood grain direction	1.5	GPa
Shear modulus of elasticity perpendicular to wood grain direction	0.8	GPa
Density of the softwood	523	kg/m^3^
Adhesive modulus of elasticity	4.6	GPa
Adhesive shear modulus	1.5	GPa
Adhesive tensile strength	40.2	MPa
Tensile strength in longitudinal direction of GFRP	393	MPa
Tensile strength in transverse direction of GFRP	23	MPa
Tensile modulus of elasticity in longitudinal direction of GFRP	32.2	GPa
Tensile modulus of elasticity in transverse direction of GFRP	5.2	GPa
Shear modulus of GFRP	3.5	GPa
Shear strength of GFRP	20.2	MPa
Poisson ratio	0.3	

**Table 2:** Geometric parameters of GFRP-softwood sandwich beams.

Specimens ^1^	a/d	L	b	d	a
		(mm)	(mm)	(mm)	(mm)
L2	2	495	90	74	165
V2	2	495	90	74	165
L4	4	945	90	74	315
V4	4	945	90	74	315
L6.5	6.5	1440	90	74	480
V6.5	6.5	1440	90	74	480

^1^ The first letter of the specimen label represents the fibre direction of the timber core: (L) longitudinal and (V) vertical. The number is the shear-span-to-depth ratio a/d.

**Table 3:** Major experimental results of the tested sandwich beam specimens.

Specimens	*(P/δ)_exp_*	Experimental Bending Stiffness *EI_exp_*	Shear Deformability	Ultimate Failure Load *P_u_*	Mid-Span Bending Moment *M_b_*
		(kN·mm^2^) × 10^8^	%	kN	kN·m
L2	18.18	1.24	38.29	101.74	8.39
V2	12.59	1.17	54.26	64.30	5.30
L4	4.07	1.48	16.89	85.98	13.54
V4	2.58	0.98	20.20	53.93	8.49
L6.5	1.13	1.31	7.24	68.25	16.38
V6.5	0.72	0.84	9.18	37.09	8.90

**Table 4:** Comparison of strain results between experimental and FE analysis.

Specimens	% Strain at Mid-Span Top			% Strain at Mid-Span Bottom			% Strain at Shear-Span		
	Exp.	FE	% D	Exp.	FE	% D	Exp.	FE	% D
L2	−0.39	−0.46	−15.22	0.42	0.45	−6.67	1.26	1.13	11.5
V2	−0.48	−0.49	−2.04	0.49	0.50	−2	0.91	0.85	7.06
L4	−0.76	−0.81	−6.17	0.72	0.81	−11.11	1.01	0.87	16.09
V4	−0.72	−0.75	−4	0.83	0.75	10.67	0.74	0.79	−6.33
L6.5	−1.02	−1.01	0.99	0.98	1.01	−2.97	0.71	0.67	5.97
V6.5	−0.78	−0.80	−2.56	0.76	0.86	−11.62	0.50	0.58	−13.8

**Table 5:** Comparison of bending stiffness and failure load results between FE analysis and experimental results.

Specimens	FE Bending Stiffness			Failure Load (kN)		
	*(P/δ)_FE_*	*EI_FE_* (kN·mm^2^) × 10^8^	% D *(EI_exp_/EI_FE_)*	Exp.	FE	% D
L2	19.11	1.33	−4.51	101.7	107.6	−5.48
V2	13.42	1.28	−8.59	64.3	73.2	−12.15
L4	3.91	1.38	7.24	86	95.8	−10.23
V4	2.66	1.01	−2.97	53.9	61.4	−12.21
L6.5	1.16	1.32	−0.76	68.2	75.5	−9.67
V6.5	0.69	0.80	5	37.1	41.0	−9.51

## Data Availability

The raw/processed data required to reproduce these findings cannot be shared at this time, as the data also form part of an ongoing study.
